# Introduction to the *Medicines* Special Issue on Acupuncture—Basic Research and Clinical Application

**DOI:** 10.3390/medicines5030099

**Published:** 2018-09-04

**Authors:** Gerhard Litscher

**Affiliations:** Research Unit for Complementary and Integrative Laser Medicine, Research Unit of Biomedical Engineering in Anesthesia and Intensive Care Medicine, and TCM Research Center Graz, Medical University of Graz, Auenbruggerplatz 39, EG19, 8036 Graz, Austria; gerhard.litscher@medunigraz.at; Tel.: +43-316-385-83907; Fax: +43-316-385-595-83907

**Keywords:** acupuncture, moxibustion, myopia, post-stroke, depression, pulse diagnosis, low back pain (LBP), osteoarthritis, uterine cancer, sciatica

## Abstract

This *Medicines* special issue focuses on the further investigation, development, and modernization of acupuncture in basic research settings, as well as in clinical applications. The special issue contains 12 articles reporting latest evidence-based results of acupuncture research, and exploring acupuncture in general. Altogether 44 authors from all over the world contributed to this special issue.

This special issue contains 12 articles with different topics in the field of modern acupuncture research (Figure 1).

The editorial “Laser Acupuncture Research: China, Austria, and Other Countries—Update 2018”contains an overview of the current status of published articles on the subject of laser acupuncture research [1].

“Acupuncture and Lifestyle Myopia in Primary School Children—Results from a Transcontinental Pilot Study Performed in Comparison to Moxibustion” [2] is the title of a prospective pilot study in 44 patients aged between 6 and 12 years with myopia. Possible therapeutic aspects with the help of evidence-based complementary methods like acupuncture or moxibustion have not yet been investigated adequately in myopic patients. This study showed that both acupuncture and moxibustion can improve myopia of young patients. Acupuncture seems to be more effective than moxibustion in treating myopia; however further Big data studies are necessary to confirm or refute the preliminary results.

“Individual Differences in Responsiveness to Acupuncture: An Exploratory Survey of Practitioner Opinion” from David Mayor et al. [3] documents patient characteristics that may influence responsiveness to acupuncture treatment, reporting results from an exploratory practitioner survey. Quantitative and qualitative analyses were then conducted. Practitioner characteristics influence their appreciation of patient characteristics. Factors consistently viewed as important included ability to relax, exercise and diet. Acupuncture practitioners may benefit from additional training in certain areas. Surveys may produce more informative results if reduced in length and complexity.

The aim of another study is to examine the short-term effect of visual function following acupuncture treatment in patients with congenital idiopathic nystagmus and acquired nystagmus [4]. Therefore an observational pilot study on six patients with confirmed diagnosis of nystagmus was performed. The applied acupuncture protocol showed improvement in the visual function of nystagmus patients and thus, in their quality of life. Further studies are mandatory to differentiate which group of nystagmus patients would benefit more from acupuncture.

Some feelings elicited by acupuncture-type interventions are “nonspecific”, interpretable as resulting from the placebo effect, our own self-healing capacities. Expectation is thought to contribute to these nonspecific effects. In the article “Nonspecific Feelings Expected and Experienced during or Immediately after Electroacupuncture: A Pilot Study in a Teaching Situation” the authors describe the use of two innovative 20-item questionnaires in a teaching situation [5]. Cluster analysis suggested the existence of two primary feeling clusters, “Relaxation” and “Alertness”. Feelings experienced during or immediately after acupuncture-type interventions may depend both on prior experience and expectation.

Post-stroke depression (PSD) is common and has a negative impact on recovery. Although many stroke patients have used acupuncture as a supplementary treatment for reducing stroke comorbidities, little research has been done on the use of acupuncture to prevent PSD. Within a contribution to this special issue [6] the authors controlled for potential confounders, and it appears that using acupuncture after a stroke lowers the risk of depression.

Radial artery (RA) pulse diagnosis has been used in traditional Asian medicine for a long time. In this article, the authors measured blood flow volume and heart rate variability in the RA and evaluated its fluctuations [7]. It is suggested that fluctuation in the volume at low frequencies of RA is influenced by the fluctuation in velocity; on the other hand, fluctuation in the volume at high frequencies is influenced by the fluctuation in vessel diameter.

In addition to the research articles there are also two review articles included in this special issue [8,9].

Within the last 10 years, the percentage of low back pain (LBP) prevalence increased by 18%. The management and high cost of LBP put a tremendous burden on the healthcare system [8]. Many risk factors have been identified, such as lifestyle, trauma, degeneration, postural impairment, and occupational related factors; however, as high as 95% of the cases of LBP are non-specific. Acupuncture for LBP is one of the most commonly used non-pharmacological pain-relieving techniques. This is due to its low adverse effects and cost-effectiveness. In this article, the causes and incidence of LBP on global health care are reviewed [8].

The most effective and safe treatment site for pain is in the skin. Another review article discusses the reasons to treat pain in the skin [9]. Pain is sensed in the skin through transient receptor potential cation channels and other receptors. These receptors have endogenous agonists (yang) and antagonists (yin) that help the body control pain.

Two case reports are also available [10,11]. Osteoarthritis is a widespread chronic disease seen as a continuum of clinical occurrences within several phases, which go from synovial inflammation and microscopic changes of bone and cartilage to painful destructive changes of all the joint structures. The first case study [10] included two patients with clinical signs of osteoarthritis and diagnosis of medial pain. The results were positive, acupuncture was effective as an alternative or complementary treatment of knee osteoarthritis, with high levels of improvement within a modest intervention period.

For women, gynecological or obstetrical disorders are second to disc prolapse as the most common cause of sciatica. As not many effective conventional treatments can be found for sciatica following uterine cancer, patients may seek assistance from complementary and alternative medicine. Here, the authors present a case of a woman with severe and chronic sciatica secondary to uterine cancer who experienced temporary relief from acupuncture [11].

Last but not least an interesting answer is given: “Why We Need Minimum Basic Requirements in Science for Acupuncture Education” [12]. Acupuncture education for both licensed physicians and non-physicians needs to include science, evidence, and critical thinking.

## Figures and Tables

**Figure 1:**
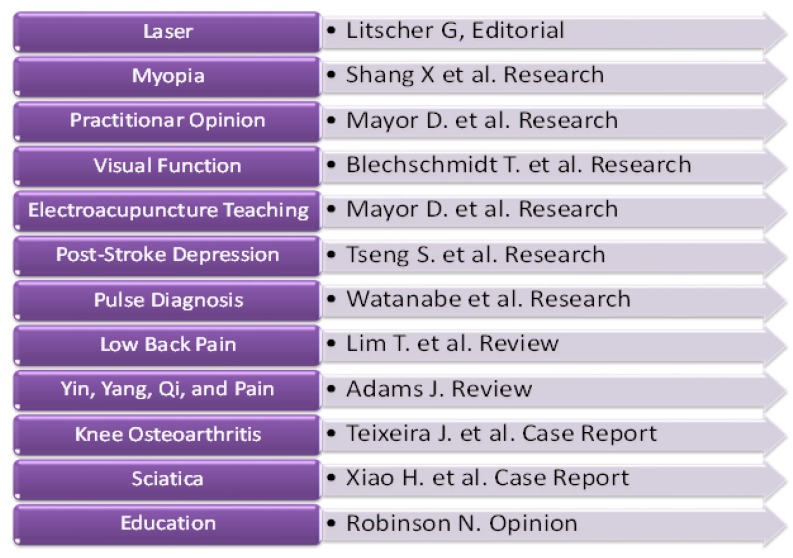
Topics of the present *Medicines* special issue.
